# A Novel Procedure for Rapid Imaging of Adult Mouse Brains with MicroCT Using Iodine-Based Contrast

**DOI:** 10.1371/journal.pone.0142974

**Published:** 2015-11-16

**Authors:** Ryan Anderson, A. Murat Maga

**Affiliations:** 1 Center for Developmental Biology and Regenerative Medicine, Seattle Children’s Research Institute, Seattle, Washington, United States of America; 2 Division of Craniofacial Medicine, Department of Pediatrics, University of Washington, Seattle, Washington, United States of America; 3 Department of Oral Biology, University of Washington, Seattle, Washington, United States of America; Wayne State University, UNITED STATES

## Abstract

High-resolution Magnetic Resonance Imaging (MRI) has been the primary modality for obtaining 3D cross-sectional anatomical information in animals for soft tissue, particularly brain. However, costs associated with MRI can be considerably high for large phenotypic screens for gross differences in the structure of the brain due to pathology and/or experimental manipulations. MicroCT (mCT), especially benchtop mCT, is becoming a common laboratory equipment with throughput rates equal or faster than any form of high-resolution MRI at lower costs. Here we explore adapting previously developed contrast based mCT to image adult mouse brains *in-situ*. We show that 2% weight per volume (w/v) iodine-potassium iodide solution can be successfully used to image adult mouse brains within 48 hours post-mortem when a structural support matrix is used. We demonstrate that hydrogel can be effectively used as a perfusant which limits the tissue shrinkage due to iodine.

## Introduction

Micro computed tomography (mCT) is an indispensable tool for acquiring high-resolution three dimensional images from mineralized tissue like bone or teeth, but has limited applicability on imaging soft tissue due to fairly low attenuation of X-rays in low density material. Magnetic Resonance Imaging (MRI) scanners with high-field-strength magnets (7 or 14T) typically provide very good contrast among soft tissues and excellent spatial resolution to image developing murine or avian embryos [1,2] as well as adults. However, they are not as widely available as research mCT scanners, and costs associated with them are generally an order of magnitude higher due to high maintenance costs and service contracts. Likewise, optical technologies like Optical Projection Tomography (OPT) or Optical Coherence Tomography (OCT) have been applied to image developing mouse and avian embryos [3–6], but the lack of commercial support for the former and the limited penetration depth of the latter modality, limits their wide adoption in the field of developmental biology. Recently, a series of methodological improvements in tissue fixation, stability and staining to improve soft tissue contrast in mCT demonstrated the feasibility of using mCT in the context of developmental biology research [7–14]. Improved soft-tissue contrast, and the lower cost of mCT instruments creates new opportunities [15,16] that were previously typically only accessible by MRI [17]. Here, we present a novel application of the tissue stabilization for iodine-based contrast agents to image adult mouse brains using mCT in which we used the previously published structural polymer matrix [18], hydrogel, as a perfusant. Our goal is to image the whole adult mouse brain in-situ using mCT, without needing to dissect it out.

## Methods

### Reagents

For tissue fixation, either 4% Paraformaldehyde (PFA) or hydrogel (Hg) was utilized. The 4% weight per volume (w/v) PFA was prepared by dissolving powdered PFA (Sigma-Aldrich, 158127) in 0.01M Phosphate Buffered Saline (PBS) at 65°C and was made fresh for perfusion. Hg solution was prepared using a previously published recipe (4% volume per volume (v/v) PFA (Sigma-Aldrich, 158127), 4% (v/v) acrylamide (Fisher Scientific, 79-06-1), 0.05% (v/v) Bis acrylamide (Boston Bioproducts, Bis-2), 0.25% (w/v) VA-044 initiator (Wako, 27776-21-2), 0.05% (w/v) Saponin (Sigma-Aldrich, 8047-15-2), 0.01M PBS, in ddH2O) [12,18] and was kept frozen until use, and then was thawed at room temperature before use.

Iodine-potassium iodide (I2KI) solution was used to stain the soft tissue because of its quick penetration and low toxicity. Although elemental iodine (I2) does not dissolve in water in any appreciable amount by itself, with the addition of potassium iodide (KI), it forms tri-iodide ions with increased solubility [7]. Thus, we prepared I2KI stain by dissolving 2% (w/v) elemental iodine (I2) (Acros, 19656) and 4% (w/v) potassium iodide (KI) (Sigma-Aldrich, 746428-800G) in Ultrapure water.

### Tissue preparation

Six C57B6/J female mice (8–9 weeks of age) underwent cardiac perfusion using a Perfusion Two (Leica AG) controlled pressure sacrifice perfusion system. Mice were first anesthetized with Isoflurane until no twitch response was obtained from a toe pinch. After sedation, each mouse was dissected from the bottom of the abdomen to the rib cage to expose the abdominal and chest cavity for transcardiac perfusion. Perfusion began with a solution consisting of 10% sucrose (w/v) in ultrapure water at 300mmHg in order to remove blood and provide disruption of the blood brain barrier to facilitate diffusion of fixative [19]. Sucrose perfusion was continued until muscle spasms ceased, about ~90–120 seconds. Then, for a period of 20 seconds, fixative, either 4%PFA or Hg, was added to the 10% sucrose solution being perfused. Perfusion pressure was then reduced to 200 mmHg and the feed tube for the 10% sucrose solution was closed. Fixative was perfused into the mice for exactly 15 minutes to allow thorough diffusion and penetration of the fixative (~45 mL total fixative). The liver was monitored during perfusion for discoloration to assess proper perfusion. Following perfusion, the mouse heads were separated from the body via decapitation several vertebrae below the base of the skull. All animal procedures in this experiment were approved by the Institutional Animal Care and Use Committee of the Seattle Children’s Research Institute.

### Baseline imaging and tissue staining

Severed heads were immediately scanned on a Skyscan 1076C (Bruker, Belgium) micro-computed tomography (mCT) scanner using an acquisition setting (0.5mm Al filter, 55kV voltage, 180uA current, 100ms exposure, 0.6 degree rotation steps, three frames per rotation step were averaged, 34.42 micron voxel size, 360° scan) optimized for skull imaging. After the baseline scan was acquired, skulls were cleaned of soft tissue, including eyes, all skin and fur, muscles, and tongue. If not removed, the stain disproportionally accumulates on the external surface of the head and interferes with the subsequent imaging. Next, the mandible, most of the snout anterior to the cribriform plate, zygomatics, orbits, and remaining vertebrae were also removed. Exposed cranial sutures were gently eroded using a micro-abrasion tool and two holes on the squamosal portion of the temporal bone were drilled to enable efficient and uniform diffusion of the stain into the endocranial space using a size 75 wire gauge drill bit. Following tissue removal the skulls were either (1) incubated at 37°C for 3 hours if they were perfused with Hg to polymerize the gel (Group A, N = 5), or (2) Incubated at room temperature in 4% PFA for 3 hours before placement in iodine stain (Group B, N = 1).

After the incubation, skulls were placed in 40mL amber borosilicate glass vials filled with I2KI to the rim and incubated at room temperature in the dark on a roller set at ~10 rotations per minute. At 24h, specimens were scanned to evaluate the penetration of the stain using scan settings that were optimized for the contrast agent (0.5mm Al filter, 70kV voltage, 140uA current, 100ms exposure, 0.6 degree rotation steps, three frames per rotation step were acquired and averaged, 34.42 micron voxel size, 360^°^ scan). At these settings, each scan took about 10 minutes. After the scans, specimens were returned to their container with fresh I2KI solution and were stained for another 24 hours. At 48 hours, skulls were taken out of stain, rinsed, and placed in a 15mL tube with PBS, then scanned a second time.

### Brain image segmentation, atlas building and comparisons

All samples were rigidly registered to their respective baseline mCT scan using DRAMMS registration library [20]. For each sample, a label map outlining the skull was created from the baseline scan using a threshold effect filter in 3D Slicer (bone grayscale value set to 35). Using this outline as the boundary, stained brains were interactively segmented using the editor module of 3D Slicer.

To compare the quality of our segmentations visually and quantitatively against high-resolution MRI, we downloaded a published C57BL/6J MRI dataset [21, 22] from http://lbam.med.jhmi.edu/. This dataset consisted of nine mixed sex individual 60 post-natal days (P60) of age. We then used the DRAMMS deformable registration in a classic unbiased population-registration framework [23] to build an atlas from the reference dataset, as well as our group A samples. The atlas construction framework iteratively finds a virtual space that resides in the centroid of the study population (i.e. the deformations needed to transform all subjects into this virtual space sum up to zero). Therefore, the constructed atlas is unbiased to any subject in the population, and is hence representative of the mean anatomy/geometry of the population [23, 24].

## Results

**Fig 1:**
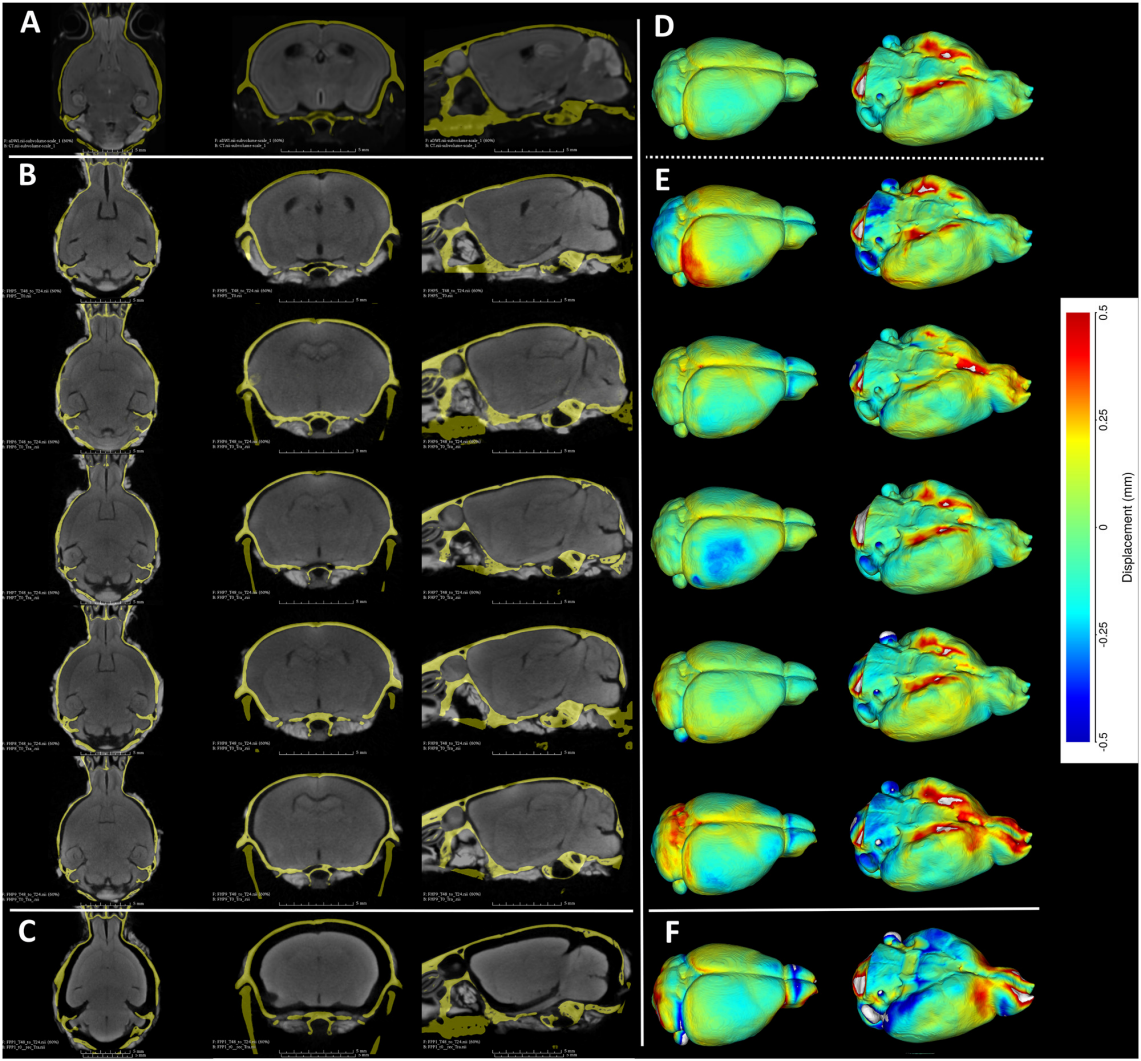
**A.** A representative high-resolution MRI scan of an age matched C57BL/6J displaying the brain and the mCT overlay (yellow outline) from [21]. **B.** Equivalent cross sections from five females that constitute the Group A (Hg perfusion) after 48h of staining in 2% I2KI. **C.** One individual that constitutes Group B (PFA perfusion) after 48h of staining in 2% I2KI. **D-F:** Heatmaps showing the difference in the obtained segmentations with respect to the reference MRI atlas constructed from [21,22]. To remove volumetric differences due to sex and age, we isometrically scaled our segmented brains to match the volume of the reference MRI atlas. Root mean square (RMS) errors are calculated after the scaling. **D:** Comparison of our Group A mCT atlas. RMS = 0.128.mm. **E:** Comparison of individuals of Group A. RMS values are 0.172mm, 0.143mm, 0.156mm, 0.142mm, 0.196mm respectively. **F:** Comparison of Group B. RMS = 0.232 mm. All comparisons are rendered on MRI reference atlas. Grey areas in the heat map indicate regions of large difference (>0.5mm) either due to extreme shrinkage or difference in segmentation.

The penetration of the stain at 24h was not sufficiently uniform to provide a good segmentation of the whole brain for either perfusion groups. By 48H, however, the brain was uniformly stained. With the apparent tissue shrinkage just starting to appear, we did not seek to stain the brains any further. The sample (Group B) perfused with PFA as the sole perfusant, displayed extreme shrinkage at 48hrs (Fig 1C). The group A samples that were perfused with Hg remained stable in most aspects of the anatomy and were most similar to the MRI imaged samples (Fig 1A) in terms of anatomical stability, with a comparable degree of staining (Fig 1B) to the PFA perfused sample.

Since our samples were slightly more variable in age (P55-P63), but more importantly female only, the result from the direct comparison would be overwhelmed by the signal from the sexual dimorphism present in the MRI sample group. Instead, we chose to uniformly scale our segmented brains to size of the reference atlas, register them to, and then finally visualize the differences in the shape as a heat map. From these heat maps, we calculated root mean square (RMS) error for each of the samples Fig 1E and 1F). The agreement between our group A atlas and the reference was very good with an RMS error of 128 (Fig 1D). The RMS error for individual samples varied between 142–196 microns.

## Discussion

The value of I2KI based stained mCT scanning was demonstrated multiple times in context of developmental biology [9,10,25]. Because iodine-based contrast agents cause significant tissue shrinkage, it is important to support tissue with a stabilizing matrix like Hg [12]. Such need is clearly demonstrated in our group B sample where there was extreme shrinkage in 48 hrs, obscuring the internal anatomy (Fig 1C). The shrinkage is also not uniform, with the regions that are exposed to the solution longest (such as around ear openings and olfactory) differentially shrunk more, as indicated by large RMS errors around those areas (Fig 1F). In case of embryos, samples are typically incubated in Hg for multiple days, after which the gel is polymerized and then simply peeled off from the surface of the embryo [26]. Such an approach is not feasible for in-situ imaging of the brain. Polymerized gel will clog all the natural openings into the endocranial space and render the diffusion uneven and unpredictable. Instead, by using Hg as a perfusant, we demonstrate that it is possible to achieve the similar level of tissue stability such that whole brains from adult mice can be stained and visualized using high-resolution mCT in detail comparable to high-resolution MRI without any significant shrinkage.

There is significant time and cost benefits for the proposed approach. From perfusion to final scan is less than 60 hrs, which is much shorter than the typical seven-day incubation necessary for typical high-resolution MRI imaging using gadolinium–diethylene triamine penta-acetic acid (Gd-DTPA). Micro CT scanning (10 minutes per scan) is much faster than MRI scanning (several hours to overnight per scan) and thus is usually less expensive than high-resolution MRI scanning. The contrast agent used, I2KI, can be prepared easily and is inexpensive. The tools necessary for manipulating the sutures are ordinary micro power tools, and a variety of them can be found in any neuroscience laboratory conducting stereotaxic surgeries. For younger samples, especially neonates, such manipulation of the cranial sutures may not be necessary. Similar to the conclusions of previous studies, the concentration and the duration of the I2KI stain needs to be adjusted based on the age and the size of the samples [7,9,15].

There are also certain downsides to using I2KI contrast based mCT scanning to obtain brain shape. We found that histological slides prepared from the cryo-embedded stained brains showed no structural detail (data not shown) and were not informative. This is, however opposite to what Wong et al (2012) observed for embryos stabilized in Hg and stained with 0.1N I2KI. Their I2KI stained embryos appeared to be compatible with standard hematoxylin-eosin staining. Thus, if obtaining cellular structure after volumetric imaging of brain is an experimental requirement, high-resolution MRI is still the only viable option. MRI with T2-weighted and DWI offers superior tissue contrast for different parts of the brain (such as cerebellum, hippocampus, cortex), enabling a more refined segmentation of brain structure. In our protocol, only the cerebellum was stained differently enough from the rest of the brain for segmentation.

In summary, proposed methodology to acquire 3D whole brain shape using microCT is best suited for rapid phenotyping applications where large numbers of samples need to be screened for gross differences in the overall brain shape (due to experimental treatment) using computational approaches such as voxel or tensor based morphometry. Because of effective imaging of skull through microCT scanning, our approach is particularly well suited for applications where the covariation of the skull and brain shape is investigated (Marcucio et al., 2011; Nieman et al., 2012).
